# Reference values for left atrial size by cardiovascular magnetic resonance in the Framingham Heart Study Offspring cohort

**DOI:** 10.1186/1532-429X-14-S1-P224

**Published:** 2012-02-01

**Authors:** Garima Arora, Philimon Gona, Carol J Salton, Susan J Blease, Christopher J O'Donnell, Warren J Manning, Michael L Chuang

**Affiliations:** 1 NHLBI's Framingham Heart Study, Framingham, MA, USA; 2 Beth Israel Deaconess Medical Center, Boston, MA, USA

## Background

Increased left atrial (LA) size is a marker for both clinical and subclinical cardiovascular disease (CVD) and is associated with excess cardiovascular risk. We sought to determine reference values for LA volume by CMR in a longitudinally followed, community-based cohort strictly free of major risk factors for LA enlargement, including: left-sided valve disease, hypertension, obesity, diabetes and clinical CVD.

## Methods

The Framingham Heart Study Offspring cohort was initiated in 1971, and study participants have undergone extensive “cycle” examinations every 3-4 years since then. In 2002-2005 a subset of Offspring underwent 1.5T CMR (Gyroscan NT, Philips), including breath-hold, ECG-gated SSFP cines in the 2- and 4-chamber (ch) views. LA borders were manually planimetered at the ventricular end-systolic phase. Care was taken to exclude the pulmonary veins and the LA appendage. LA volume was calculated as V=0.85*A2ch*A4ch/Lmin (A=planimetered area, Lmin is the shorter of 2- and 4-ch lengths). Mean±SD and 95th percentile upper limits [95%UL] were determined; two-sample t-test was used to compare men with women for LA volume, and 2- and 4-ch cross-sectional areas and lengths. LA volume was also indexed to height (HT), HT2.7 and body surface area (BSA). Clinical covariates used in this study ranged from 1971 through cycle 7 (1998-2001). For generation of LA reference values, we prospectively excluded Offspring with any history of hypertension (SBP>140 or DBP>90 mmHg), obesity (BMI≥30 kg/m2), clinical CVD, diabetes or left-sided murmurs on any cycle visit.

## Results

Of 1471 datasets analyzed, 839 were excluded due to presence (or any history) of risk factors for LA enlargement, leaving 257 men and 375 women for analysis. Ages ranged from 37 to 88 years, with mean (±SD) age of 62±9 years, p=NS for men vs. women. Table 1 shows unindexed and indexed LA volumes by sex. Table 2 shows unindexed LA areas and lengths. Men had greater raw LA volume, a difference that was maintained with indexation to HT, but was attenuated after indexation to HT2.7 and to BSA. LA area and length were greater in men than women.

**Table 1:** Reference Values for Left Atrial Volume

Mean±SD [95%UL]	Men, N=257	Women, N=375	Men vs. Women
Volume, ml	79±22 [115]	65±19 [98]	P<0.001
Volume/HT, ml/m	45±12 [66]	40±11 [59]	P<0.001
Volume/ HT2.7	17.1±4.7 [25.5]	17.6±4.9 [26.5]	P=0.19 (NS)
Volume/BSA, ml/m2	40±11 [59]	38±11 [57]	P=0.15 (NS)

**Table 2:** Left Atrial Cross-Sectional Areas and Lengths

Mean±SD [95%UL]	Men, N=257	Women, N=375	Men vs. Women
4-ch area, cm2	20.6±4.1 [27.6]	18.9±4.0 [25.6]	P<0.001
2-ch area, cm2	21.3±4.6 [29.2]	18.4±4.1 [24.3]	P<0.001
4-ch length, mm	50.8±7.1 [62.9]	49.2±6.5 [60.1]	P=0.004
2-ch length, mm	50.8±6.9 [62.2]	47.4±6.8 [58.1]	P<0.001

## Conclusions

We report sex-specific reference values for LA volume determined using SSFP CMR in a community-dwelling population of clinically relevant age who were strictly free of factors commonly associated with LA enlargement. Men have greater native LA volume than women, but indexation to HT2.7 or to BSA attenuates sex differences in LA volume.

## Funding

NIH/NHLBI contracts N01-HC-25195 and RO1 HL70279.

